# Role of previous parity in the relationship between lifetime physical activity and later life cognition: The Rancho Bernardo study

**DOI:** 10.1177/13872877261477010

**Published:** 2026-08-12

**Authors:** Vanja Petrović, Emilie T. Reas, Raylene A. Reimer, Cindy K. Barha

**Affiliations:** 1 Faculty of Kinesiology, 2129 University of Calgary, Calgary, Alberta, Canada; 2 Department of Neurosciences, 8784 University of California, San Diego, La Jolla, CA, USA; 3 Department of Biochemistry and Molecular Biology, Cumming School of Medicine, 2129 University of Calgary, Calgary, Alberta, Canada; 4 Hotchkiss Brain Institute, 2129 University of Calgary, Calgary, Alberta, Canada

**Keywords:** Alzheimer's disease, cognition, parity, pregnancy history, physical activity, postmenopause

## Abstract

**Background:**

Parity history influences dementia risk and cognitive aging, and recent evidence suggests it may also influence the association between physical activity and cognition in later life.

**Objective:**

To examine associations between total lifetime and life-stage–specific physical activity and later life cognition in postmenopausal females with differing parity histories.

**Methods:**

This cross-sectional analysis using data from the Rancho Bernardo Study included 867 postmenopausal females with complete data, categorized into three parity groups (number of pregnancies >6-months): nulliparous, 1–2 pregnancies, and grand-multiparous (≥3 pregnancies). Cognitive outcomes included executive functions and memory. Physical activity was assessed using a self-report questionnaire capturing retrospective activity during adolescence, age 30, age 50, and current activity in later life. Covariates included age, education, health composite score, body mass index, and hysterectomy status. Linear models examined associations between physical activity and domain-specific later life cognitive outcomes stratified by parity.

**Results:**

Greater total lifetime physical activity was associated with higher executive functions in nulliparous and grand-multiparous females. Moderate activity in nulliparous females and high activity in grand-multiparous females during adolescence and at age 30 were associated with higher executive functions. High physical activity at age 50 and currently was associated with higher executive functions in nulliparous females.

**Conclusions:**

The findings suggest the relationship between self-reported physical activity and cognition was strongest in the two groups at greater risk for cognitive decline and Alzheimer's disease, the nulliparous and grand-multiparous groups. Further research is needed to understand the mechanisms driving parity differences.

## Introduction

### Background and rationale

With a new case diagnosed every three seconds, dementia is becoming a leading cause of death and disability worldwide.^1^ Females seem to be at the center of this disease, as they are two to three times more likely than males to be diagnosed with Alzheimer's disease (AD), the most prevalent type of dementia accounting for 60–70% of all cases.^2,3^ Females also show greater cognitive decline after AD diagnosis and greater AD-related neuropathology.^4,5^ These significant sex differences in prevalence seem to be related to female-specific reproductive events, which are hypothesized to influence brain health and trajectories of age-associated cognitive decline.^6^ Specifically, menopause has been proposed as a driver of brain aging. Menopause marks a critical stage in a female's life, as it is associated with dramatic declines in estradiol (E2)^7^ and the loss of its neuroprotective effects, which has profound implications for female brain aging. Additionally, parity history is another female-specific factor that has been linked to altered brain health and disease risk.^6^ However, only a limited number of studies have examined the long-term effects of these sex-specific factors on female brain health, and the evidence is still far from conclusive.

Parity-related hormonal changes and their interaction with immune processes are hypothesized to affect maternal brain neuroplasticity and structure, potentially influencing the trajectory of cognitive and brain aging later in life.^8,9^ Growing evidence suggests that parity history is associated with AD risk and cognitive decline, with effects dependent on the specific number of births.^6,10–13^ Grand multiparity, defined as having three or more complete pregnancies or, in some studies, defined as five or more, has been linked with a higher risk of AD, earlier age of onset, and higher brain levels of amyloid.^10,13–15^ One of the studies examining the relationship between grand multiparity and brain abnormalities associated with AD found that grand multiparity was associated with atrophy or shrinkage of the hippocampus, a strong biomarker and predictor of AD.^16,17^ Although the specific mechanisms triggering atrophy are poorly understood, researchers have proposed that repeated stress and changes in the level of E2 caused by multiple pregnancies may be important contributors.^16^ However, grand-multiparous females are not the only parity group at higher risk of cognitive decline and dementia. Although much less studied, nulliparity, defined as having no completed pregnancies, is also associated with a higher risk of AD after the onset of menopause.^18,19^ Current evidence suggests that nulliparous females are more likely to undergo early menopause (defined as having final menstrual period between the ages of 40–44 years), resulting in a significant drop in E2 levels earlier in life, thereby increasing the risk of chronic diseases as they age, including dementia.^15,20,21^ Together, these findings suggest that nulliparity and parity greater than a certain number, especially three or more, could increase the risk of AD, highlighting the importance of considering parity history when studying female brain health in older age.

Physical activity is a recognized modifiable lifestyle factor supporting brain health in older age,^22^ and emerging evidence indicates that its cognitive benefits may be moderated by parity history.^11,23,24^ The long-term effects of parity on neuroplasticity and brain health may influence the capacity of the female brain to respond to physical activity later in life. Interestingly, using data from the Health, Aging, and Body Composition Study, Barha and colleagues found that females with different parity histories have different cognitive responses to physical activity.^11^ Their secondary analyses of a cohort study showed that grand-multiparous and nulliparous older females, those at higher risk of cognitive impairment and AD, exhibited a stronger positive association between cognitive performance and level of self-reported walking.^11^ Results of a recent cross-sectional study on the association between physical activity and cognition in postmenopausal females with different parity histories further support this, demonstrating stronger associations between cognition and physical activity in the high-parity (defined as three or more pregnancies) group compared to the low-parity groups.^23^ Previous work by Reas and colleagues suggests that physical activity levels across the lifespan and during specific life stages are important to consider within the context of later life cognition.^25,26^ Findings from the Rancho Bernardo Study^25^ show that regular physical activity across the lifespan was associated with better late-life function in multiple cognitive domains (i.e., global cognition, executive functions, and episodic memory) and reduced odds of cognitive impairment, with older age physical activity showing the strongest associations. They argued that physical activity in youth and midlife is also important because it may contribute to cognitive reserve and modify associations in later years. Taking these findings into account, examining whether the amount and/or timing of physical activity across the lifespan differentially relate to later life cognition depending on parity status would aid in the development of tailored exercise interventions for females at higher risk of cognitive decline.

### Objective

The objective of the present study was to explore the relationship between physical activity and domain-specific cognition in older postmenopausal females as a function of parity history by conducting a secondary analysis of existing data from the Rancho Bernardo Study (RBS), a longitudinal population-based study of healthy aging. We hypothesized stronger positive relationships between lifetime frequency of physical activity and later life cognition among the nulliparous and grand-multiparous females. This will be specifically seen for the domain of executive functions, as this cognitive domain has been shown to be most responsive to physical activity and exercise.^27–29^ Additionally, we hypothesized that physical activity at different points throughout the lifespan would show different strengths of associations with later life cognition across these two parity groups.

## Methods

### Study design

This study was a cross-sectional analysis of data collected as part of the Rancho Bernardo Study (RBS), a population-based study of healthy aging established between 1972 and 1974 in the Southern California community of Rancho Bernardo.

### Participants

The present study consists of 867 females who participated in the 1988–1992 (study visit 5) assessments at the Rancho Bernardo Study research clinic. The study sample was limited to participants who had complete pregnancy history, physical activity, and cognitive data. The majority of participants were White of northern European descent, middle to upper-middle class, and highly educated, with over 60% having at least some post-secondary education. Participants’ vital status was monitored annually through mailed surveys, and they were invited to return for clinical follow-up every 4 years, completing up to 12 visits. During these clinic visits, comprehensive assessments were conducted, including sex-specific health history, health status, health behaviours, physical function, medication use, and blood samples. The Human Research Protections Program of the University of California in San Diego approved this study. Each participant provided written informed consent prior to each clinic visit.

### Variables

*Demographics and covariates*. Demographics were collected via self-administered questionnaires at study visit 5. For the present analyses, age, education, health-related information, hysterectomy status (surgical removal of the uterus), and body mass index (BMI; kg/m^2^) were utilized. The highest level of education obtained was split into five categories: <12 years of education, 12 years, 13–15 years, 16 years, and >17 years of education. Participants reported whether they had been diagnosed (yes/no) with the following health conditions: stroke, heart attack, congestive heart failure (CHF), diabetes, high blood pressure, transient ischemic attack (TIA), high cholesterol, angina, pulmonary disease, liver disease, kidney disease, thyroid disease, arthritis, osteoarthritis, Parkinson disease, and any type of cancer. A composite score was created that reflected the total number of these health conditions an individual had.

*Parity*. From 1988 to 1992 (visit 5), pregnancy history (parity) information was collected via a self-administered sex-specific questionnaire. Parity was defined as the number of births lasting at least 6 months of gestation, and births were reported as live. Based on prior literature,^10,30,31^ participants were stratified into three parity groups [nulliparous (0 births), low parity (1–2 births), grand-multiparous (≥3 births)].

*Physical activity*. Physical activity was quantified at visit 5 using a modified version of the Godin Leisure-Time Exercise Questionnaire (GLTEQ).^32^ Participants were asked to report their frequency (number of times during the typical 7-day period) and intensity (mild = minimal effort, moderate = not exhausting, strenuous = heart beats rapidly) of physical activity engagement in the past (teenage, 30 years of age, 50 years of age) and currently. To examine the lifetime frequency of physical activity engagement per week, a continuous total lifetime physical activity index was created by averaging the number of physical activity bouts engaged in per week across all life stages (teenage, at age 30, at age 50, current), with higher values indicating greater overall activity. Second, an energy expenditure score was computed as previously done,^32^ taking into account the intensity and frequency of physical activity using the equation: (9 × Strenuous) + (5 × Moderate) + (3 × Mild). Scores were calculated separately for each life stage (teenage, age 30, age 50, and current), and participants were classified into the three physical activity groups using established cutoffs^32^: highly active (≥24 units), moderately active (14–23 units), and sedentary (<14). This was done to examine physical activity engagement and energy expenditure across physical activity intensities at different life stages.

*Cognition*. Several cognitive tests were administered at study visit 5.^33^ We chose to focus on the domains of executive functions and episodic memory for the following reasons: 1) physical activity and exercise show the strongest effects for the domain of executive functions^34^; 2) episodic memory shows greater declines with aging in females^35^; and 3) the role of episodic memory decline in AD.^36^ Executive functions were measured with the Trail Making Test, Part B (TMT-B) and the Verbal Fluency Test (VFT). TMT-B, originally part of the Halstead-Reitan Neuropsychological Test Battery, is a test of executive function that assesses set-shifting and visuomotor tracking.^37,38^ Participants are required to draw lines sequentially in order to connect 25 circles that contain both numbers and letters in ascending order (e.g., 1, A, 2, B, 3, C, etc.). Scores are based on the number of seconds required to finish the test, with higher scores (seconds) indicating poorer set-shifting performance. The VFT also measures executive functions by asking participants to generate as many words as they can from a given semantic category in one minute.^39^ The retrieval of lexically associated words from long-term memory and frontal lobe-controlled processes, which are considered as higher-level executive functions, are essential for completing this test.^40^ The Buschke Selective Reminding Test (BSRT) assesses verbal episodic learning and memory and involves six trials where a person is presented with a list of words and asked to recall them immediately after they were presented.^41^ After a short-term recall attempt, the participant is reminded of the words that were not recalled on that particular trial. “Selective reminding” continues across the remaining trials, allowing analysis of participants’ verbal learning and long-term retrieval.^42^ The Visual Reproduction test (VRT) measures visual episodic memory and comprises the VRT-I, which assesses immediate recall and the VRT-II, which assesses delayed recall.^43^

Separate cognitive domain composites were created for executive functions and episodic memory by standardizing each test with z-scores. For each separate cognitive test, raw scores were z-standardized based on the analytic sample and then averaged within each cognitive domain to form a composite. The executive functions composite was comprised of TMT-B (reverse-coded) and VFT, as these are among the seven most frequently used executive functions tests in the literature.^44^ The memory composite comprised the Buschke Short-term Recall (BSTR) short-term recall, long-term recall, and total recall, and the VRT-I and VRT-II. Higher composite values indicate better cognitive performance.

### Bias

Parity and physical activity were assessed retrospectively using self-reported questionnaires. The risk of substantial recall bias for parity is considered relatively low because pregnancy is a major and salient life event that is generally likely to be recalled accurately. In contrast, retrospective reporting of physical activity across the life course may be more susceptible to recall bias. Participants may have had difficulty accurately recalling the frequency, duration, and intensity of physical activity engaged in many years earlier, which could have resulted in exposure misclassification. Cognitive outcomes were assessed using standardized cognitive tests rather than self-reported cognitive functioning. However, recall, social desirability, and measurement bias cannot be excluded and should be considered when interpreting the findings.

### Statistical analysis

All statistical analyses were performed using R version 4.2.1 (https://www.r-project.org) and R Studio (https://www.rstudio.com/), a recommended integrated development environment.^45^ Only complete cases were included, thus statistical analyses examining the role of parity in the physical activity-cognition relationship included 867 females.

Demographic and visit 5 participants’ characteristics were examined by parity group (nulliparous (0 births), low parity (1–2 births), grand-multiparous (≥3 births)) using ANOVA and chi-square tests. Due to limitations with sample size and reduced power to detect interactions,^46^ parity-stratified linear regressions were conducted to examine whether the pattern of the relationship between total lifetime physical activity engagement and cognitive-domain-specific performance (executive functions composite, memory composite) differs among the three parity groups. Only in those parity groups that showed a significant relationship did we further examine whether the frequency and intensity of physical activity at different life stages (teenage, age 30, age 50, and current) mattered by examining the main effect of physical activity status based on energy expenditure score (highly active, moderately active, sedentary) on the cognitive composite of interest using linear regressions. Age, education, health composite, hysterectomy status, and BMI were used as covariates in all analyses. As these are exploratory, hypothesis-generating analyses, alpha was set at 0.05, giving us a 95% confidence level.

## Results

### Descriptive data

**Figure 1.**
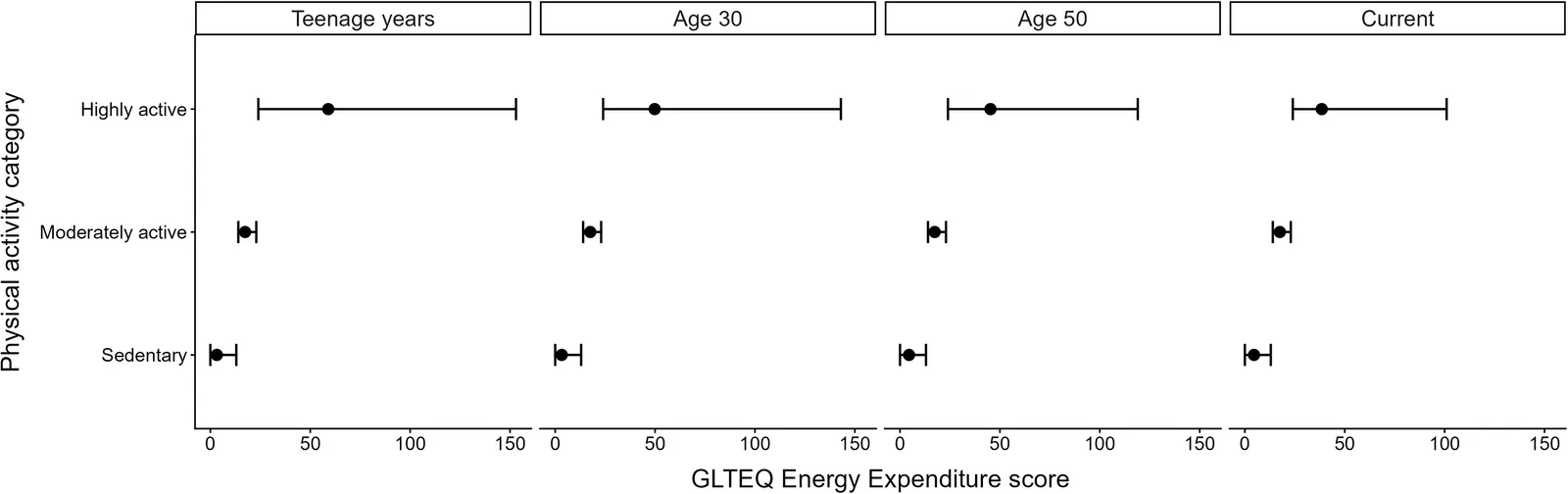
Mean GLTEQ energy expenditure scores and observed ranges by physical activity category and life stage. Points represent mean scores, numerical labels indicate the corresponding means, and vertical lines represent the observed minimum and maximum values.

Table 1 shows the visit 5 characteristics of the sample of 867 females as a function of parity group (nulliparous (0 births), 1–2 births, and grand-multiparous (≥3 births)). Between-group differences were found for age, health status, education, BMI, and several cognitive tests. The nulliparous group was slightly older, more frequently in the highest education category, and reported the fewest health conditions compared with the other two groups. The nulliparous group also had the lowest mean BMI and visit 5 scores on TMT-B, VFT, total and long-term recall and/or recognition components of the BSTR, and VRT, while the grand-multiparous group had the highest mean BMI and scores on all tests except the short-recall component of the BSTR. The proportion of participants with a hysterectomy did not differ significantly across groups. Table 2 shows that parity groups did not differ in their self-reported physical activity engagement, except for mild and moderate physical activity during the teenage period. The distribution of participants across the sedentary, moderately active, and highly active categories did not differ significantly between groups at any assessed life stage. Figure 1 shows the mean GLTEQ Energy Expenditure score and observed score range for each physical activity category across the four examined life stages.

**Table 1.** Participant characteristics for each parity group separately at study visit 5.

Mean (*SD*) or *N* (%)	Nulliparous	1–2 pregnancies	Grand-multiparous	*p*
N	199	391	277	
Age	75.4 (8.7)	73.6 (9.1)	68.2 (9)	<0.001*
Education				<0.001*
<12 years	7 (3.5%)	21 (5.4%)	6 (2.2%)	
12 years	68 (34.2%)	135 (34.5%)	87 (31.4%)	
13–15 years	69 (34.7%)	133 (34%)	113 (40.8%)	
16 years	38 (19.1%)	78 (19.9%)	55 (19.9%)	
17 + years	17 (8.5%)	24 (6.1%)	16 (5.8%)	
Health composite	1.5 (1.1)	1.8 (1)	2 (1.1)	<0.001*
Hysterectomy				0.142
1 (no)	99 (50.8%)	192 (49.5%)	158 (57%)	
2 (yes)	96 (49.2%)	196 (50.5%)	119 (43%)	
Body Mass Index (BMI)	24.1 (3.9)	24.5 (3.6)	25.2 (4.5)	0.005*
Executive Functions				
TMT-B time (s)	139.6 (61.2)	138.7 (67.6)	119.3 (58.5)	<0.001*
Verbal fluency	17 (4.7)	17.8 (4.8)	18.4 (4.7)	0.007*
Executive Functions z-score	−0.1 (0.8)	0 (0.8)	0.2 (0.8)	<0.001*
Memory				
BTR score	39.6 (8.4)	40.2 (9.1)	42 (8.7)	0.006*
BSTR score	5.8 (3.9)	5.9 (4.3)	5.1 (3.9)	0.033*
BLTR score	33.8 (11.4)	34.4 (12.4)	36.8 (11.9)	0.009*
BWR score	9.9 (0.4)	10 (0.3)	10 (0.2)	0.247
VRT-I score	9.3 (3.4)	9.5 (3.5)	10.4 (3.5)	<0.001*
VRT-II score	6.5 (3.9)	6.8 (4)	7.8 (4)	<0.001*
Memory z-score	0 (0.5)	0 (0.5)	0.1 (0.5)	<0.001*

*statistically significant; TRAIL-B: Trail Making Test, Part B; VFT: Verbal Fluency Test; BTR: Buschke Total Recall from 6 trials; BSTR: Buschke Short-term Recall; BLTR: Buschke Long-term Recall; BWR: Buschke Word Recognition Total; VRT-I: Visual Reproduction Test (immediate recall); VRT-II: Visual Reproduction Test (delayed recall). Higher scores on all these tests indicate better performance.

**Table 2.** Physical activity variables for each parity group.

Mean (*SD*) or *N* (%)	Nulliparous	1–2 pregnancies	Grand-multiparous	*p*
N	199	391	277	
Mild Physical Activity (bouts/week)				
Mild PA current	2.6 (2.3)	2.6 (2.3)	2.5 (2.3)	0.726
Mild PA age 50	2.5 (2.4)	2.4 (2.4)	2.4 (2.3)	0.935
Mild PA age 30	2 (2.4)	2 (2.6)	2.1 (2.6)	0.746
Mild PA teen	1.9 (2.7)	2.4 (3)	2.7 (3.1)	0.015*
Moderate Physical Activity (bouts/week)				
Moderate PA current	1.3 (2.1)	1.6 (2.3)	1.7 (2.3)	0.145
Moderate PA age 50	1.6 (2.2)	1.8 (2.2)	1.9 (2.3)	0.283
Moderate PA age 30	1.8 (2.3)	2 (2.4)	2 (2.4)	0.418
Moderate PA teen	2.9 (2.7)	3.5 (2.8)	3.6 (2.7)	0.013*
Strenuous Physical Activity (bouts/week)				
Strenuous PA current	0.2 (0.8)	0.2 (0.8)	0.2 (0.8)	0.785
Strenuous PA age 50	0.4 (1.3)	0.5 (1.3)	0.5 (1.5)	0.608
Strenuous PA age 30	0.5 (1.5)	0.6 (1.4)	0.6 (1.6)	0.503
Strenuous PA teen	1.7 (2.4)	2 (2.5)	2 (2.4)	0.392
Overall lifetime PA (avg)	1.6 (1.1)	1.8 (1.3)	1.9 (1.3)	0.082
Physical Activity Status (Teenage; Energy Expenditure score, units)				0.064
Sedentary	45 (22.6%)	90 (23%)	50 (18.1%)	
Moderately active	36 (18.1%)	42 (10.7%)	39 (14.1%)	
Highly Active	118 (59.3%)	259 (66.2%)	188 (67.9%)	
Physical Activity Status (Age 30; Energy Expenditure score, units)				0.446
Sedentary	101 (50.8%)	191 (48.8%)	126 (45.5%)	
Moderately active	42 (21.1%)	70 (17.9%)	51 (18.4%)	
Highly Active	56 (28.1%)	130 (33.2%)	100 (36.1%)	
Physical Activity Status (Age 50; Energy Expenditure score, units)				0.509
Sedentary	99 (49.7%)	185 (47.3%)	120 (43.3%)	
Moderately active	43 (21.6%)	77 (19.7%)	57 (20.6%)	
Highly Active	57 (28.6%)	129 (33%)	100 (36.1%)	
Physical Activity Status (Current; Energy Expenditure score, units)				0.554
Sedentary	97 (48.7%)	176 (45%)	120 (43.3%)	
Moderately active	57 (28.6%)	111 (28.4%)	75 (27.1%)	
Highly Active	45 (22.6%)	104 (26.6%)	82 (29.6%)	

*statistically significant; PA: physical activity.

### Parity-dependent relationship between total lifetime physical activity and later life cognition

**Figure 2.**
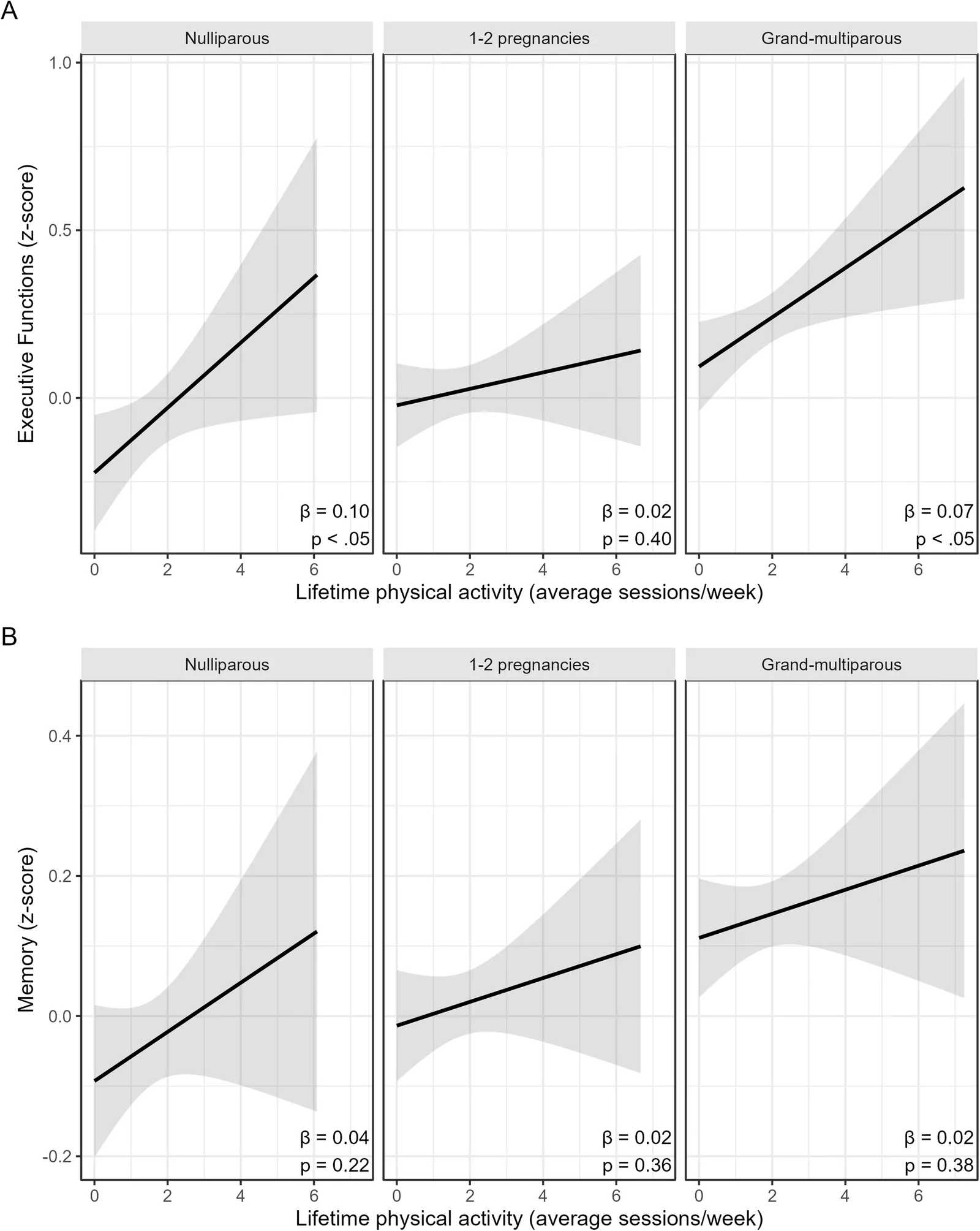
Associations between average number of physical activity sessions engaged in per week across the lifespan and late-life domain-specific cognition within each parity group. (A) Higher executive functions scores were associated with greater lifetime physical activity in nulliparous and grand-multiparous females. (B) Episodic memory scores were not associated with lifetime physical activity in any parity group. *Notes:* Lines represent predicted cognitive z-scores from parity-stratified linear models, with shaded bands indicating 95% confidence intervals. Models were adjusted for age, education, health composite, hysterectomy status, and BMI. β coefficients and *p*-values for lifetime physical activity are shown within each panel.

Stratified analyses were conducted to examine the relationship between total lifetime physical activity engagement and cognition within each parity group separately. Greater total lifetime physical activity engagement was significantly associated with higher executive functions composite scores in the nulliparous (β = 0.10, 95% CI [0.007, 0.187], *p* = 0.033) and grand-multiparous (β = 0.07, 95% CI [0.013, 0.134], *p* = 0.017) groups (see Figure 2A), but not in the 1–2 pregnancy group (β = 0.02, 95% CI [-0.033, 0.082], *p* = 0.400). No significant associations were observed for the memory composite in any of the parity groups (β ranged from 0.02 to 0.04, all *p* > 0.05; see Figure 2B).

### Parity-dependent life stage relationship between physical activity status and later life executive functions

Subsequent analyses were restricted to nulliparous and grand-multiparous females, as these were the only groups in which total lifetime physical activity engagement showed a significant association with executive functions. Thus, within the nulliparous and grand-multiparous groups, additional analyses examined whether physical activity status (highly active, moderately active, sedentary) at specific life stages contributed to later life executive functions.

**Figure 3.**
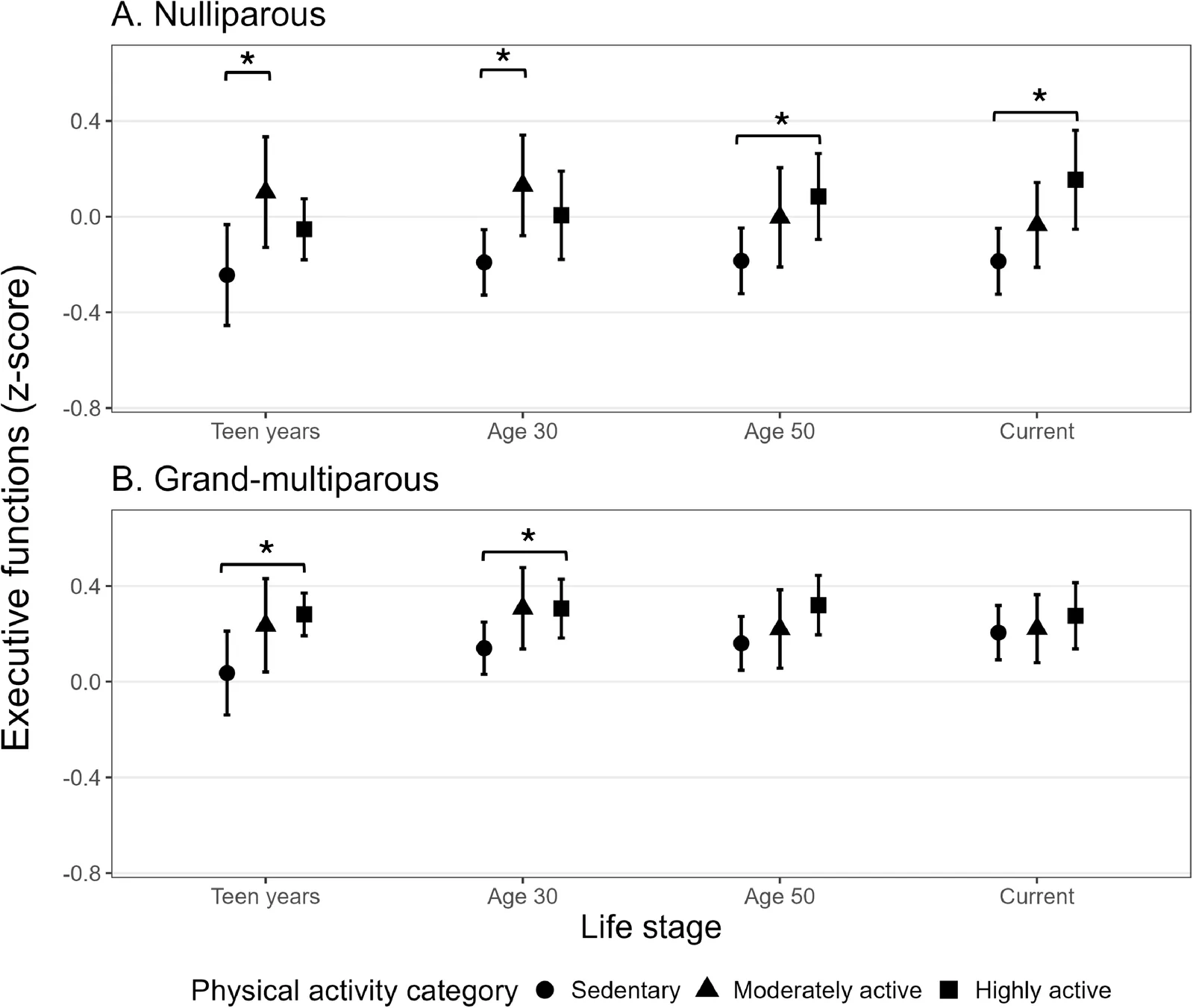
Associations between physical activity status (sedentary, moderately active, highly active) and later life executive functions composite scores across the four life stages (teenage years, age 30, age 50, current age) within nulliparous females (A) and grand-multiparous females (B). *Notes:* Points represent adjusted mean cognitive z-scores, and error bars represent 95% confidence intervals. Models were adjusted for age, education, health composite, hysterectomy status, and BMI. The sedentary group serves as the reference group.

*Teenage life stage*. In the teenage life stage, parity-stratified analyses revealed that compared with the sedentary nulliparous females, the moderately active nulliparous females exhibited significantly higher executive functions scores (β = 0.35, 95% CI [-0.035, 0.657], *p* = 0.029), whereas the highly active nulliparous females did not differ (β = 0.19, 95% CI [-0.062, 0.445], *p* = 0.138; Figure 3A). In the grand-multiparous group, compared with the sedentary females, the highly active females showed significantly higher executive functions scores (β = 0.25, 95% CI [0.046, 0.445], *p* = 0.016), whereas the moderately active females did not differ (β = 0.20, 95% CI [-0.062, 0.461], *p* = 0.135; Figure 3B).

*Age 30 life stage*. At age 30, parity-stratified analyses revealed that compared with the sedentary nulliparous females, the moderately active nulliparous females exhibited significantly higher executive functions scores (β = 0.32, 95% CI [0.070, 0.574], *p* = 0.012), whereas the highly active nulliparous females did not differ (β = 0.20, 95% CI [-0.038, 0.431], *p* = 0.100; Figure 3A). In the grand-multiparous group, compared with the sedentary females, the highly active females showed significantly higher executive functions scores (β = 0.17, 95% CI [-0.001, 0.332], *p* = 0.051), whereas the moderately active females did not differ (β = 0.17, 95% CI [-0.036, 0.370], p = 0.107; Figure 3B).

*Age 50 life stage*. At age 50, parity-stratified analyses revealed that compared with the sedentary nulliparous females, the highly active nulliparous females demonstrated significantly higher executive functions scores (β = 0.27, 95% CI [0.040, 0.498], *p* = 0.02), whereas the moderately active nulliparous females did not differ (β = 0.18, 95% CI [-0.069, 0.432], *p* = 0.154; Figure 3A). In the grand-multiparous group, compared with the sedentary females, the highly active females showed a trend towards higher executive functions scores (β = 0.16, 95% CI [-0.009, 0.329], *p* = 0.065; Figure 3B).

*Current life stage*. At the current time point, parity-stratified analyses revealed that compared with the sedentary nulliparous females, the highly active nulliparous females exhibited significantly higher executive functions (β = 0.34, 95% CI [0.088, 0.593], *p* = 0.008), whereas the moderately active nulliparous females did not differ (β = 0.15, 95% CI [-0.074, 0.377], *p* = 0.188; Figure 3A). Within the grand-multiparous group, the moderately active and highly active females did not differ from the sedentary females (β ranged from 0.02 to 0.08, all *p* > 0.05; Figure 3B).

## Discussion

### Key results

Our results indicate that the associations between self-reported lifetime physical activity engagement and later life domain-specific cognitive performance differ between parity groups. Specifically, we found that greater total lifetime physical activity engagement was associated with higher executive functions in nulliparous and grand-multiparous females. Total lifetime physical activity was not associated with cognition in the 1–2 pregnancy group across cognitive domains. When examining the timing of physical activity engagement across the lifespan as a predictor of later life cognition, we found that being moderately active in nulliparous females and highly active in grand-multiparous females during the teenage years and at age 30 were associated with higher executive functions. At age 50, being highly active for nulliparous females was associated with higher executive functions, while in grand-multiparous females, there was a trend toward significance. Interestingly, only in nulliparous females was being currently highly active associated with higher executive functions. These findings suggest greater protective effects of physical activity in nulliparous and grand-multiparous females, particularly for the cognitive domain of executive functions, and that grand-multiparous females may need to be more active earlier in life to reap the protective effects later in life.

The findings of this study suggest that the positive relationship between physical activity engagement and cognitive performance in later life among females is most pronounced in the two parity groups most at risk of AD,^13^ nulliparous and grand-multiparous. This was specifically seen for the cognitive domain of executive functions, which is one of the first and most severely affected cognitive domains with aging.^34,44^ Previous randomized controlled trials suggest that physical activity has its greatest benefits for the domain of executive functions,^28^ with older females showing larger effect sizes than older males.^29^ Our findings are consistent with previously reported associations between physical activity and later life cognition in nulliparous and grand-multiparous groups.^11,23^ For example, Barha and colleagues^11^ assessed self-reported walking (minutes/week) over a 10-year period and found that greater average walking engagement was associated with higher average global cognition and average executive functions, but only among nulliparous and grand-multiparous females. Additionally, higher average walking was associated with less decline in global cognition and executive functions over 10 years in only grand-multiparous females. Similarly, Chen and colleagues^23^ reported that physical activity, defined as engagement in common exercise types (e.g., walking, cycling, swimming, dancing) for more than 20 min at least once per week, was associated with higher global cognition among postmenopausal females with high parity (≥3 births). Although further research is needed, these findings together suggest that cognitive function in grand-multiparous and nulliparous females is more responsive to physical activity throughout the lifespan than in those with medium parity (e.g., 1–2 pregnancies).

Parity history influences cognitive aging trajectories,^9,47–49^ with studies indicating that grand multiparity and nulliparity are associated with lower cognitive performance, greater cognitive decline over time, and greater AD risk.^13,16,18,50–52^ While the mechanisms underlying these associations are not fully understood, parity has long-lasting effects on neuroplasticity, including increases in hippocampal neurogenesis and synaptogenesis as seen in rodent models,^53^ which have important implications for brain health and cognitive ageing.^8,11,54–56^ These parity effects on neuroplasticity and cognition are believed to be partly related to hormonal fluctuations and accompanied by changes in immune function;^8^ however, other underlying mechanisms remain unknown, making it unclear how specific parity histories may, in turn, influence the relationship between physical activity and cognition in older age. Given that parity history shapes neuroplastic levels in the aging brain,^57,58^ it may be that physical activity, which also influences the same neuroplastic markers,^59^ interacts with parity to further influence cognition.

Long-lasting effects of parity on cardiovascular disease could help partly explain the association we observed between high physical activity and better executive functions in grand-multiparous and nulliparous females. Many, but not all, studies show that both grand-multiparity^60–63^ and nulliparity^60,62,64,65^ seem to be associated with increased risk of cardiovascular disease. Furthermore, grand-multiparity has been associated with well-established cardiovascular disease risk factors (e.g., greater waist and hip circumference, hypertension, metabolic changes),^66,67^ all of which can be attenuated by engaging in physical activity.^68,69^ Discrepancies between studies could be related to differences in how grand-multiparity was defined (studies ranged from 3 to ≥6 births).^60–63^ As well, most of these studies examining the association between parity and cardiovascular disease risk used small sample sizes and did not adjust for pregnancy complications (e.g., hypertension, preeclampsia, diabetes); thus, we must be cautious in interpreting these findings.

Additionally, parity alters age-related changes in central and peripheral inflammation,^8^ and previous work implicates inflammation in cognitive aging.^53,70^ A rodent study examining short- and long-term effects of primiparity vs. nulliparity on hippocampal neurogenesis and aging-related changes in the profile of circulating cytokines found overall that nulliparity was associated with greater aging-related changes in inflammatory signaling.^53^ For example, they found that circulating pro-inflammatory cytokines such as IFN-y and IL-10 increased with age in nulliparous but not primiparous rats. In humans, cross-sectional analyses have shown that grand-multiparity is related to elevated levels of CRP, IL-12 and TNF inflammatory markers.^71–73^ Therefore, the potential anti-inflammatory effects of physical activity, which include lowering pro-inflammatory markers such as TNF and CRP while increasing anti-inflammatory IL-6,^74^ may help partly explain the associations we observed in nulliparous and grand-multiparous females.

The findings of this study also suggest that the relationship between physical activity status and executive functions differed by life stage in which the activity was happening. In general, we found that engaging in at least moderate levels of physical activity during the teenage years, and at ages 30 and 50 was beneficial for later life executive functions in nulliparous females and being highly active earlier in life was needed to see a benefit to cognition in grand-multiparous females. Current evidence indicates that physical activity earlier in life has long-lasting protective effects on brain function because it stimulates neurogenesis, neuronal growth, and production of neurotrophic factors.^75–77^ Findings from a retrospective study^78^ of postmenopausal females suggest that moderate physical activity (an estimation of the weekly frequency of moderate activity) beginning during teen years is associated with better performance across several cognitive measures in later life (e.g., global cognition, executive functions, processing speed, and working memory). Another study of postmenopausal females found that those who reported being physically active earlier in life had considerably lower odds of late-life cognitive impairment.^79^

Similarly, Reas and colleagues^25^ found that teenage physical activity protects against age-related cognitive decline. They reported that greater teenage physical activity engagement was associated with higher global cognition scores in females, and also that teenage physical activity modified the relationship between physical activity and executive functions later in life. Specifically, they examined interactions between teenage and older age physical activity on cognitive performance and found that active older individuals performed better on executive functions tests if they were also active as teenagers, whereas no such benefit was seen for those inactive as teenagers. Additionally, they found that greater physical activity at age 30 was associated with better executive functions test scores.^25^ Together, this evidence supports the role of early-life physical activity as a promising factor for long-term cognitive health in females.

### Limitations

Although the findings of this study are consistent with the hypothesis that there are different cognitive responses to physical activity across parity groups, several limitations should be considered. This was a cross-sectional study of a relatively small sample of predominantly white, educated, and relatively healthy middle-class older adults, which means that causal inferences cannot be made and observed patterns of physical activity-cognition associations across parity groups cannot be generalized to more diverse or less healthy cohorts. However, our homogeneous sample minimizes confounding by race, socioeconomic status, and health care access. Although age was included as a covariate in our analyses, differences in age at the time of cognitive assessment between the three parity groups could have influenced the observed associations between physical activity and cognitive function.

Our physical activity variables were derived from a widely used, valid self-report measure of physical activity that combined retrospective recall of earlier life stages (teenage, 30 s, 50 s) with current assessments in later life, potentially leading to recall and social desirability bias. This may also have reduced our ability to detect modest interaction effects. Accordingly, given the smaller sample size and the limitations with the physical activity measure, we were underpowered to reliably detect interaction effects of parity and physical activity on late-life cognitive outcomes; thus, the parity-stratified analyses should be regarded as hypothesis-generating and require confirmation in larger studies. Moreover, physical activity was assessed at specific life stages (teen, 30, 50, current) rather than continuously across the lifespan. Changes in activity between these time points were therefore not captured (e.g., ages 31–49), which may have limited our ability to identify long-term physical activity patterns. Additionally, our finding that lower physical activity was associated with lower cognition may be challenged by reverse causation, as poorer cognitive health may have contributed to lower activity engagement earlier in life.^80^ As well, reduced memory may have affected the accuracy of participants’ retrospective recall of physical activity engagement (e.g., in those with cognitive or memory impairment). Therefore, a more objective and repeated prospective assessment of physical activity would likely improve measurement accuracy and enhance confidence in the observed associations.

Except for parity and hysterectomy, other reproductive variables (e.g., age at menopause, duration of reproductive lifespan and/or age at first and last pregnancy) were available for only a small portion of female participants in this cohort; thus, statistically controlling for these variables would have reduced our sample size considerably. Additionally, factors like depression and social connection may have influenced the observed association between physical activity and cognition across different parity groups. Depression is linked to poorer cognitive performance and may also differ between parity groups.^81^ Additionally, depression is associated with lower physical activity levels in older adults.^82^ Thus, if depression was more common among nulliparous or grand-multiparous females, some of the lower cognitive scores observed in these groups may have been related to depression and not only parity itself. Similarly, social connection could have also influenced the findings since greater social connection may be associated with both higher physical activity and cognitive outcomes.^83^ Therefore, it is important to acknowledge that the unmeasured factors may have resulted in residual confounding, although the direction of this bias is uncertain and may differ across parity groups. Taking all of this into account, the findings of this study are considered preliminary and hypothesis-generating and should be interpreted with caution. Replication in a more diverse female cohort with objectively quantified physical activity levels will be important to confirm these parity-dependent associations between physical activity and later life cognition.

### Conclusion

The current study provides preliminary evidence that the association between physical activity throughout the lifespan and later life cognition differs across parity groups. Specifically, our results suggest stronger positive associations between total lifetime physical activity and executive functions among the grand-multiparous and nulliparous females, two groups that are at greater risk of AD and cognitive impairment. Additionally, our life-stage-specific results suggest that being active or moderately active during teen years and at age 30 is associated with better later life executive functions among the nulliparous and grand-multiparous groups. Being active at age 50 and later relates to better executive functions among the nulliparous group. Together, these findings show that parity history may be relevant to understanding variability in cognitive aging trajectories among females and that an active lifestyle may be a promising strategy for maintaining brain health into older age, which can in turn reduce dementia burden.
